# Genome-wide association study reveals the genetic basis of vitamin C content in rapeseed (*Brassica napus* L.) seedlings

**DOI:** 10.3389/fpls.2025.1649023

**Published:** 2025-07-23

**Authors:** Chao Wang, Lieqiong Kuang, Ze Tian, Xinfa Wang, Hanzhong Wang, Xiaoling Dun

**Affiliations:** ^1^ Key Laboratory of Biology and Genetic Improvement of Oil Crops of the Ministry of Agriculture and Rural Affairs, Oil Crops Research Institute of the Chinese Academy of Agricultural Sciences, Wuhan, China; ^2^ Hubei Hongshan Laboratory, Wuhan, China

**Keywords:** rapeseed seedlings, vitamin C, GWAS, candidate genes, germplasm resource

## Abstract

Rapeseed (*Brassica napus* L.) is a versatile crop, with its seedlings and flowering stalks can be utilized as vegetables, which are rich in vitamin C (Vc) and other essential nutrients, including selenium, calcium, zinc, and various amino acids. Despite the well-documented health benefits of Vc as a critical antioxidant nutrient, the genetic mechanisms governing Vc accumulation in rapeseed remain poorly understood. In this study, we investigated the Vc content of 327 rapeseed accessions during the seedling stage across six environments in Hubei province over three consecutive years (2018-2020). The Vc content in these environments ranged from 62.82 to 161.25 mg/100g, demonstrating high genetic variation (7.96% to 9.43%) and heritability (86.11%). Genome-wide association studies (GWAS) identified 31 significant single nucleotide polymorphisms (SNPs) across various chromosomes, which explained 5.68% to 12.78% of the phenotypic variation, integrated into 16 quantitative trait loci (QTLs). Kyoto Encyclopedia of Genes and Genomes (KEGG) and Gene Ontology (GO) enrichment analyses of the 2365 annotated genes near associated SNPs revealed significant involvement in diverse metabolic pathways including peroxisome, ascorbate, secondary metabolites, and terpenoid biosynthesis, as well as biological processes such as hydrogen peroxide/lactate metabolism and ROS biosynthesis, along with associations with specific cellular components and molecular functions. Furthermore, we identified six candidate genes that exhibit significant differences in expression between low and high Vc accessions, which are potentially involved in Vc biosynthesis but require further experimental validation. Additionally, we selected four superior germplasms (8S079, 8S200, 8S242, and 8S243) that demonstrate extreme Vc content, providing valuable germplasm resources for breeding. Collectively, these findings provide novel and comprehensive insights into the genetic and molecular mechanisms regulating Vc accumulation in rapeseed, thereby establishing a foundation for targeted genetic improvement of nutritional quality in vegetable rapeseed varieties.

## Introduction

Rapeseed *(Brassica napus* L., AACC, 2n = 38) ranks as the third-largest oilseed crop cultivated globally, providing a significant source of nutritious and well-balanced edible oil for human consumption, while also serving as a crucial primary protein source in animal feed (Zou et al., 2019). The cultivation of rapeseed, which is integral to the global oilseed industry, is heavily influenced by global market prices, as farmers frequently make decisions driven by profit margins (Liu et al., 2022). In China, the profitability of rapeseed cultivation is increasingly undermined by rising labor costs and escalating agricultural input expenses, leading to a reduction in the area under cultivation. Without an increase in the value of rapeseed, the land allocated for its cultivation will probably keep diminishing, which could threaten the production of edible oils. Therefore, it is essential to maximize both the current and potential benefits of rapeseed.

*Brassica napus* is a relatively recent allotetraploid species that originated from the hybridization of the diploid ancestors *B. rapa* (2n = 20, AA) and *B. oleracea* (2n = 18, CC) (An et al., 2019). These two species belong to the *Brassica* genus within the *Cruciferae* family and are cultivated in China as seasonal leafy and moss vegetables. *Brassicales* is known for a diverse range of compounds that promote health, especially acknowledged for being abundant in Vc (Domínguez-Perles et al., 2014). Numerous studies have demonstrated that diets abundant in cruciferous vegetables are associated with a reduced risk of developing various forms of cancer (Li et al., 2022). These findings have contributed to the classification of cruciferous vegetables as functional foods, resulting in the availability of various dietary supplements that contain extracts or compounds derived from these vegetables in the market. Except as an edible oil, rapeseed can also serve purposes such as vegetable production, energy generation, forage, green fertilizer, and honey crop (Fu et al., 2016). The introduction of “double-low” rapeseed varieties, characterized by low levels of erucic acid and glucosinolate, has significantly improved the flavor, quality, and nutritional content of vegetable rapeseed, facilitating its dual use as both a source of oil and food. Recently, rapeseed has gained popularity as a new vegetable crop, particularly in China, where its edible seedlings and flower stalks are highly valued. In recent years, rapeseed breeding experts have selected and bred several new varieties of rapeseed flower stalks for vegetables, which are nutrient-rich, crisp, tender in texture, and flavorful. As a commonly available seasonal vegetable in winter, rapeseed flower stalks present significant economic potential. Our previous investigations have demonstrated that rapeseed seedlings and flowering stalks are abundant in vitamin C (Vc) and other essential nutrients (Zhan et al., 2024; Wang et al., 2025). Identifying the superior quality of rapeseed and breeding varieties that are high in nutrition and flavor holds substantial theoretical and practical implications for the multifunctional utilization of rapeseed.

L-Ascorbic acid (AsA), commonly referred to as Vc, is a water-soluble vitamin that serves as a crucial antioxidant molecule in both plant and animal metabolism (Fenech et al., 2019). In humans, Vc is essential for numerous physiological functions that are necessary for sustaining overall health, including the absorption of iron, the prevention of osteoporosis, the synthesis of collagen, immune system stimulation, the prevention of scurvy, epigenetic regulation, enhancement of skin quality, and the improvement of human immunity (Paciolla et al., 2019; Nowak, 2021). High dietary consumption of Vc is associated with important health benefits for consumers, including a reduced incidence of several significant human diseases and disorders (Agius et al., 2003; Meščić Macan et al., 2019). In higher plants, Vc acts not only as an antioxidant but also as a cofactor for enzymes, which is essential for several physiological functions, such as photoprotection, cell growth and division, ethylene production, and reactions to abiotic stressors (Hu et al., 2016; Mellidou et al., 2021; Zheng et al., 2022; Wu et al., 2024). It has been established that Vc is a crucial molecule for human health that cannot be synthesized endogenously (Terzaghi and De Tullio, 2022). As a result, identifying sufficient dietary sources of Vc has emerged as a significant focus within the field of nutritional research. The primary sources of Vc are fresh fruits and vegetables; thus, enhancing its concentration is expected to have a substantial impact on humans (Bulley and Laing, 2016).

The synthesis and metabolism pathways of Vc in *Arabidopsis thaliana* have established a solid foundation for analyzing Vc genetics and regulatory mechanisms in other plants. The synthesis pathway of Vc in plants is relatively complex, mainly including four distinct pathways: the L-galactose pathway, the L-gulose pathway, the D-galacturonate pathway, and the Myo-inositol pathway (Li X. et al., 2019). Currently, there is a growing focus on enhancing Vc content, not just to enhance the quality of fruits and vegetables but also to produce crops with higher stress resilience. It has been confirmed that *SlGMP3* (Zhang et al., 2013) and *AeGMP2* (Liao et al., 2021) are involved in the synthesis of Vc tomato and kiwifruit, respectively. Research has shown that overexpression of the *GME* gene can considerably enhance Vc levels and boost the stress resilience of plants (Imai et al., 2012; Ma et al., 2014). The *AcGPP* gene in kiwifruit exhibits regulation that is influenced by light conditions and various abiotic stresses, playing a crucial role in the management of Vc content (Li et al., 2012). In addition, key genes such as *APX*, *MDHAR*, and *DHAR* have been demonstrated to be involved in the Vc degradation and regeneration pathways (Liao et al., 2023).

Genome-wide association studies (GWAS) represent a crucial approach for investigating complex quantitative traits, as they utilize natural populations as study subjects and do not necessitate the construction of specific populations. This method facilitates the identification of several candidate genes linked to these traits, thereby enhancing our understanding of the genetic influences involved in complex phenotypes. GWAS has been extensively applied in the genetic analysis of rice (Zhong et al., 2021), rapeseed (Wang et al., 2018), maize (Ma et al., 2021), wheat (Yang et al., 2020), soybeans (Zhang et al., 2022), cotton (Sun et al., 2017) and others. In recent years, the nutritional benefits of rapeseed as a novel vegetable have garnered increased research attention several quality traits have been well-studied. A study that analyzed the genetic basis of soluble solids content in rapeseed flower stalks by quantitative trait loci (QTL) mapping and GWAS, providing important insights for breeding rapeseed flower stalks with high soluble solids content (Wu et al., 2021). Gao et al. (2023) identified the QTLs and potential candidate genes linked to the glucosinolates biosynthesis pathway in rapeseed flower stalks by GWAS, providing new insights into the biosynthesis of glucosinolates in flower stalk tissues. Shi et al. (2024) analyzed the QTLs and potential candidate genes related to crude fiber in the flower stalks of rapeseed by GWAS, providing fresh perspectives on the genetic underpinnings of crude fiber and contributes valuable germplasm resources for enhancing the quality of rapeseed flower stalks. The study of these traits is crucial for enhancing the marketability and consumer acceptance of rapeseed as a versatile vegetable.

In recent years, rapeseed breeding experts have developed several new varieties of rapeseed flower stalks for vegetables that are nutrient-rich, crisp, tender, and flavorful. Following the harvesting of rapeseed flower stalks and subsequent topdressing, there is a notable increase in oilseed yield, which can even result in multiple harvests simultaneously, thereby effectively augmenting the commercial value of rapeseed (Shi et al., 2023). Although several QTLs and genes linked to Vc regulation have been recognized and characterized in crops, few studies have focused on the content of Vc in rapeseed, and the molecular mechanism that underlies the production of these components is not fully understood. An in-depth understanding of the genetic mechanisms underlying Vc metabolism in rapeseed, coupled with the application of modern molecular breeding technology, will lay the foundation for cultivating rapeseed varieties that are high in Vc and rich in other nutritional elements. The objectives of this study are: (1) to examine the Vc content of 337 diverse rapeseed accessions in six environments; (2) to use the GWAS analysis to explore the genetic loci and functional genes that control the Vc content of rapeseed; (3) to identify and select superior germplasm resources that possess high Vc content, foundation for cultivating excellent germplasm of vegetable rapeseed with high-Vc content. These findings provide insights into the genetic and molecular mechanisms that influence Vc accumulation in rapeseed, highlighting the interaction between genetic factors and environmental conditions. We have identified key genetic loci and pathways associated with Vc accumulation, which can inform strategies for genetic improvement. This research supports efforts to develop vegetable rapeseed varieties with higher nutritional content, potentially enhancing human health.

## Materials and methods

### Plant materials and growth conditions

The study’s natural populations comprised 327 diverse rapeseed accessions, including 102 spring ecotypes, 191 semi-winter ecotypes, and 34 winter ecotypes, thereby highlighting the variety within the species under investigation. The accessions were sourced from the Rapeseed Genetics and Breeding Innovation Team at the Oil Crop Research Institute of the Chinese Academy of Agricultural Sciences (Wuhan, China), with the origin and composition of this population previously detailed in our earlier studies (Ibrahim et al., 2021). Field experiments were conducted across six environments, where the 327 accessions were cultivated in Yangluo (30.71°N, 114.51°E) during 2018, 2019, and 2020; in Wuchang (30.57°N, 114.33°E) in 2019 and 2020; and in Jingzhou (30.23°N, 112.34°E) in 2018, all located within Hubei province. The six environments are designated as 2018YL, 2018JZ, 2019YL, 2019WC, 2020YL, and 2020WC. A randomized complete block design, incorporating three biological replications, was utilized for field planting. Additionally, agricultural field management was executed in accordance with the standard farming practices advocated in the local region. The sampling of rapeseed seedlings occurred when they had developed five expanded leaves, approximately 30 days post-sowing. During sampling, roots and yellow leaves were removed from the rapeseed plants, and five plants from each accession that were similar in size and growth status were selected. To ensure the preservation of Vc and inhibit oxidative degradation, the rapeseed seedlings were promptly frozen in liquid nitrogen immediately following their harvest.

### Determination of Vc content

The analytical procedure for measuring Vc content adhered to previously established experimental workflows (Wang et al., 2024). Rapeseed seedlings, preserved in liquid nitrogen, were finely milled using a grinder. Accurately weighed 1 g (with a precision of 0.1 mg) of the powdered rapeseed seedlings was placed into a 50 mL centrifuge tube. Next, added 25 mL of the extraction solution, consisting of a 1% hydrochloric acid formulation, and a high shear dispersing emulsifier (ANGNI, China) was utilized to thoroughly homogenize and crush the sample. Subsequently, the supernatants were filtered using a filter with pore sizes of 0.45 μm and 0.22 μm, and a volume of 10.0 μL of the filtered supernatant was injected into the e2695 HPLC system (Waters, USA) on a monomeric C18 column (250 mm × 4.6 mm × 5 μm, CNW, China), with detection occurring at a wavelength of 245 nm. The mobile phase consisted of a mixture of methanol and 20 mM ammonium acetate in a ratio of 3:97 (v/v). The flow rate was maintained at 1.0 mL/min, the temperature was kept at 30°C, and the observed retention time was 2.67 minutes. Standard curves for Vc were established to provide reliable references for quantifying the Vc content in the analyzed samples.

### Genome-wide association study

The genotypes of 337 rapeseed accessions were analyzed using the *Brassica* 50K SNP array (Greenfafa, Wuhan). The genotyping data were processed with Illumina Bead Studio software to identify effective single nucleotide polymorphisms (SNPs) based on the following criteria: a missing rate of ≤ 0.2, a heterozygous rate of ≤ 0.2, and a minor allele frequency of > 0.05. Extensive information regarding SNP genotypes was obtained from previous studies (Ibrahim et al., 2021). A comprehensive analysis was performed, resulting in the identification of 21,242 SNPs selected for further investigation. To determine the precise physical locations of these SNP markers, the corresponding probe sequences were aligned with the *Brassica napus* reference genome “Darmor-bzh”. The association analysis was carried out using TASSEL 5 software (Bradbury et al., 2007). A mixed linear model (MLM) was employed, accounting for both the structure matrix and the relative kinship among the samples. The significance threshold for determining significantly associated SNP markers was established at *P* < 4.71 × 10^−5^ (*P* = 1/21242, – log_10_ (*p*) = 4.33). The R package lme4 was utilized to compute the best linear unbiased prediction (BLUP) for the traits (Yang et al., 2020). Additionally, the “CMplot” package in R software (R 4.3.2) was utilized to generate Manhattan and quantile-quantile (QQ) plots.

### Identification of candidate genes

The genes situated within a 500 kb range both upstream and downstream of the significant SNP loci were chosen as potential candidate genes, considering the decline of linkage disequilibrium (LD) (Chen et al., 2018; Li S. et al., 2019). The candidate genes’ functions were examined by integrating the annotation data from the “Darmor-bzh” reference genome and homologous genes from *A. thaliana*. To identify the primary biological functions associated with the candidate genes, we carried out Kyoto Encyclopedia of Genes and Genomes (KEGG) and Gene Ontology (GO) pathway enrichment analyses.

### Real-time quantitative PCR analysis

Two accessions, 8S007 and 8S243, characterized by low and high average Vc content, respectively, were selected for qRT-PCR analysis. These rapeseed accessions with extreme Vc content were grown in greenhouse hydroponics as described in our previous study (Wang et al., 2024). Following a growth duration of 16 days, samples representing the above-ground tissues of rapeseed seedlings were gathered for analysis. Total RNA was extracted from the rapeseed leaves using an RNA Extraction Kit (Omega, USA), from the rapeseed leaves. Subsequently, qRT-PCR was performed employing the ChamQ Universal SYBR qPCR Master Mix (Vazyme, China) on the instrument LightCycler^®^ 480 II Real-Time PCR System (Roche, Switzerland). For each sample analyzed in this study, four biological replicates were utilized to ensure the reliability and reproducibility of the results. The relative expression levels of the target genes in *B. napus* were calculated using the 2^−ΔΔCt^ method with *ACTIN7* employed as an internal control to normalize the data. All primer sequences utilized in this research can be found in **Supplementary Table S1**.

### Statistical analysis

The frequency distributions of Vc content assessed across six different environments were created utilizing Origin 2024b software (Origin Laboratory, USA), and the correlation coefficient was determined. The calculation of broad-sense heritability was performed using the R package lme4 (Chen et al., 2018). Statistical significance is determined by Student’s t-test or ANOVA analysis with GraphPad Prism 9 software. In the figures, various notations are employed to indicate statistical significance: *, *P* < 0.05; **, *P* < 0.01; ***, *P* < 0.001, and ****, *P* < 0.0001. Additionally, the figures display different letters above the bars to illustrate the significance groupings based on ANOVA analyses, with a threshold set at *P* < 0.05. The data presented in the figures reflect mean values, while error bars represent the standard deviation (SD) of those means.

## Results

### Phenotypic variation of Vc content among rapeseed seedlings

**Figure 1:**
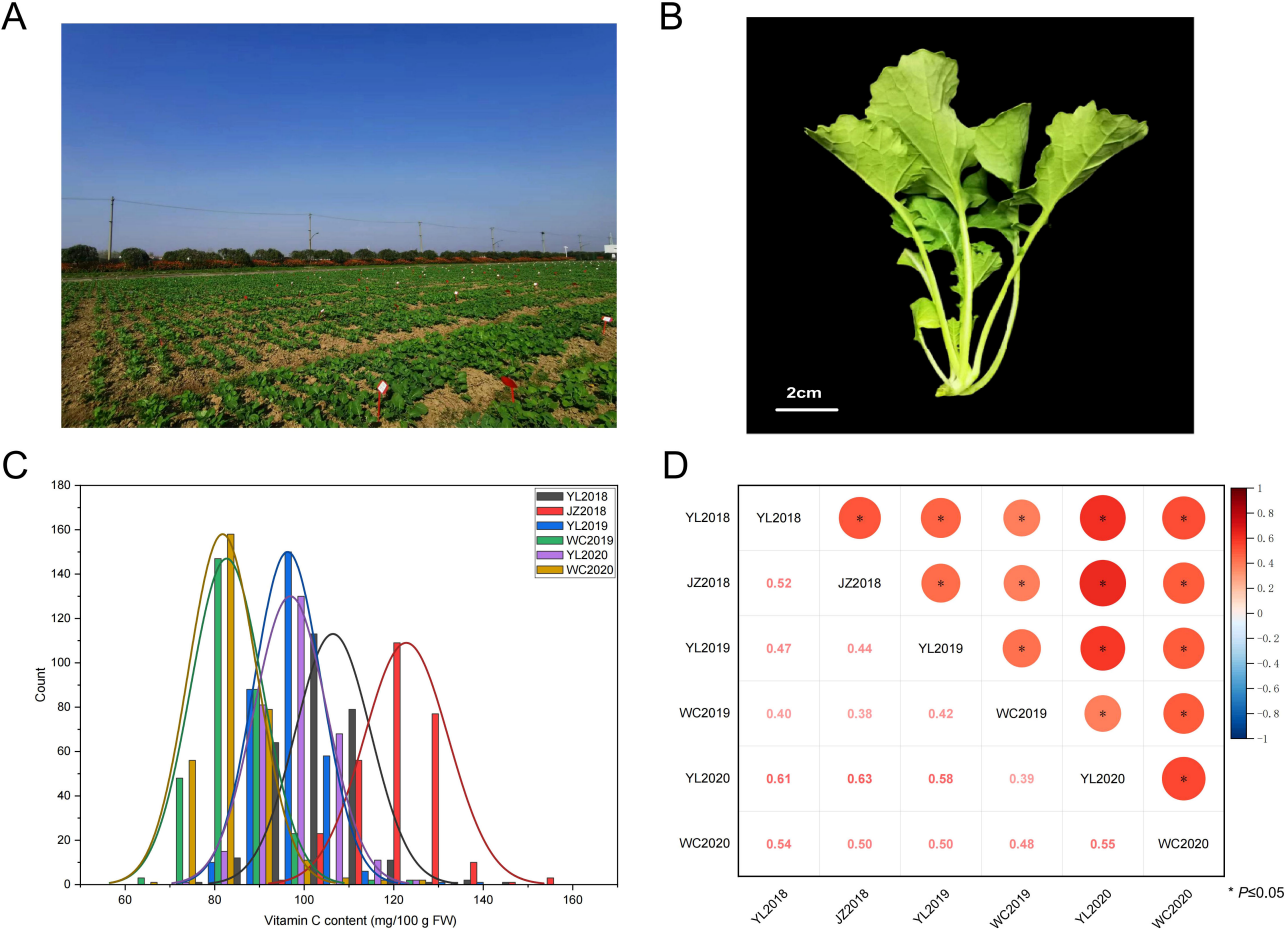
The distribution of phenotypic traits and the relationship of Vc content among 327 accessions evaluated in six environments. **(A)** Phenotypic picture of field growth in natural populations. **(B)** Sampling of rapeseed seedlings. **(C)** The variation of Vc content observed in the six environments (2018YL, 2018JZ, 2019YL, 2019WC, 2020YL, and 2020WC). **(D)** Correlation analysis of Vc content in six environments.

In order to assess the variation of Vc among the rapeseed germplasm members, we analyzed the Vc content in seedlings from 327 accessions using HPLC across six different environments (YL2018, JZ2018, YL2019, WC2019, YL2020, and WC2020). **Figures 1A, B** illustrate the field planting and sampling diagram of rapeseed seedlings, respectively. The frequency distribution analysis of Vc content across the 327 accessions revealed a distribution pattern that is either normal or closely approximates a normal distribution, as illustrated in **Figure 1C**. This suggests that the Vc content is governed by multiple genes, which is characteristic of quantitative traits, and indicates its suitability for GWAS analysis. A notable correlation was observed in all Vc contents across the six environments, with correlation coefficients ranging between 0.38 and 0.63 (*P* < 0.05) (**Figure 1D**). **Table 1** provides a comprehensive overview of the descriptive statistics regarding the Vc content of the GWAS population across the six environments. Overall, the analysis revealed that the Vc content among the 327 accessions exhibited considerable variability, ranging from 62.82 to 161.25 mg/100g across the six investigated environments, displaying 1.7 to 2.0-fold variations. The variation coefficient varied between 7.96% and 9.43% across the six environments, suggesting significant genetic diversity within the population. The broad-sense heritability (*h^2^*) was determined to be 86.11%, indicating that genetic factors primarily influenced the Vc content in the population. However, analysis of variance revealed that various factors, including genotype, environment, and the interaction between genotype and environment, all had noteworthy impacts on the six environments that were studied (**Supplementary Table S2**).

**Table 1:** Phenotypic variation of Vc content of 327 accessions in six environments.

Environment	Minimum (mg/100g)	Maximum (mg/100g)	Mean (mg/100g)	SD	CV (%)	Skewness	Kurtosis	Heritability
YL2018	80.38	144.72	106.42	8.47	7.96	0.53	2.28	86.11%
JZ2018	94.72	161.25	122.80	9.43	7.68	0.38	1.94	
YL2019	79.00	136.43	96.30	7.54	7.83	0.83	2.89	
WC2019	62.82	126.12	82.68	8.04	9.72	1.33	5.30	
YL2020	74.85	132.95	96.90	8.12	8.38	0.53	1.60	
WC2020	65.57	123.71	81.77	7.71	9.43	1.73	6.69	

### GWAS of Vc content in the association population

**Figure 2:**
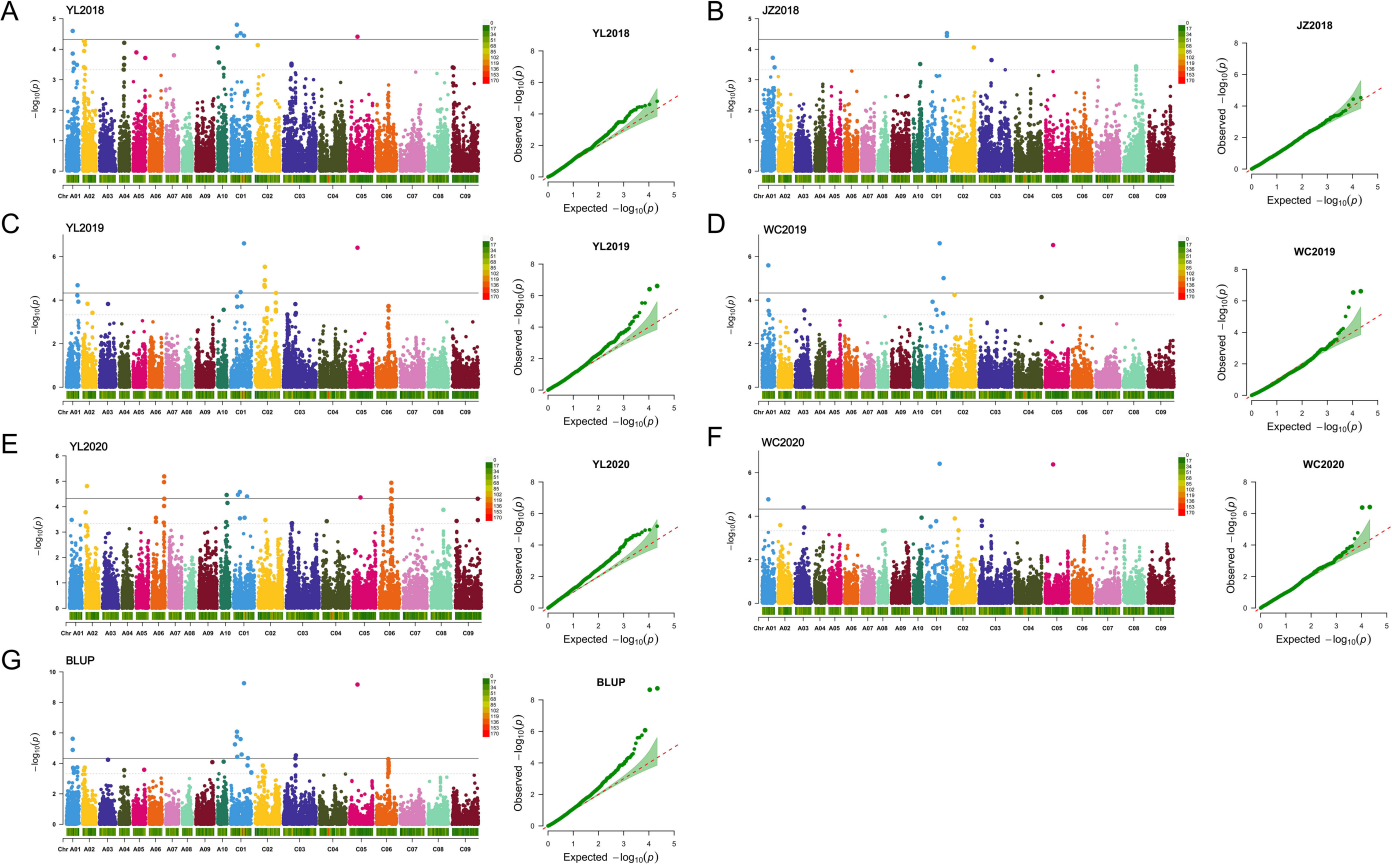
GWAS of Vc content in rapeseed seedlings. Manhattan plot and QQ plot of Vc content across six environments [**(A)** 2018YL, **(B)** 2018JZ, **(C)** 2019YL, **(D)** 2019WC, **(E)** 2020YL, **(F)** 2020WC] and the best linear unbiased prediction (BLUP) **(G)**, generated using a multivariate linear model (MLM). Within the Manhattan plot, the solid horizontal line signifies the significance threshold value adjusted using Bonferroni correction [–log_10_ (p) = 4.33].

To explore the genetic factors influencing Vc content in rapeseed seedlings, we performed a GWAS in the association population comprising 327 accessions. The Vc content across six environments and the BLUP values were calculated using the MLM model, which was selected as the optimal model by incorporating kinship correction and population structure. The Manhattan plot and QQ plot in **Figure 2** illustrate the significant SNPs associated with Vc content. As a result, the MLM model identified 31 SNPs that showed significant associations, located on chromosomes A01, A02, A03, A06, A10, C01, C02, C03, C05, and C06, explaining 5.68% to 12.78% phenotypic variation (**Supplementary Table S3**). Among these associated SNPs, 6, 2, 9, 4, 13, and 4 were identified in 2018YL (**Figure 2A**), 2018JZ (**Figure 2B**), 2019YL (**Figure 2C**), 2019WC (**Figure 2D**), 2020YL (**Figure 2E**), and 2020WC (**Figure 2F**), respectively; a total of 13 SNPs were detected through the use of BLUP values in the six environments (**Figure 2G**). It should be noted that 25.8% (8/31) significantly associated SNPs could be consistently identified across various environments (including BLUP). Specifically, three SNPs with significant associations were identified in two environments, two in three environments, and another two SNPs were found in both five environments and the BLUP, indicating a high level of reliability.

**Figure 3:**
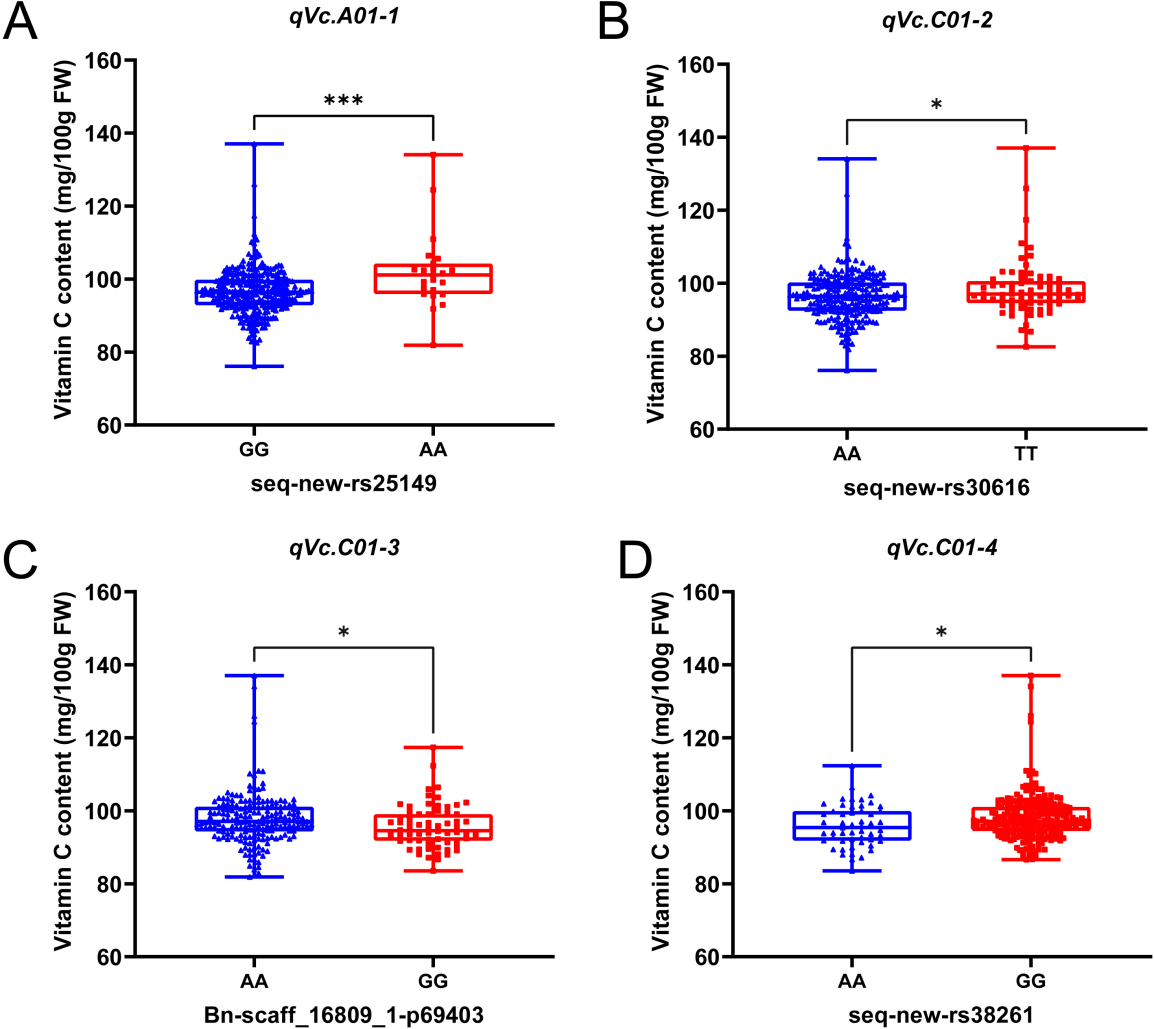
Analysis of the importance of phenotypic variation at four reproducible association loci **(A-D)**. The horizontal axis delineates the haplotype associated with the peak SNP at the identified loci of association. On the vertical axis, the content of Vc is represented. The violin plot presented illustrates both the average and the variability of Vc content corresponding to each haplotype. * and *** indicate statistically significant differences, with a P-value threshold of less than 0.05 and 0.001 respectively, as assessed through Student’s t-test.

To effectively integrate the significant clustered SNP loci, we employed a methodology documented in prior research (Chen et al., 2018; Li S. et al., 2019). In our analysis, SNPs were classified as belonging to the same loci if the lead SNP and any subsequent SNPs were located within a distance of 500 kb or exhibited an LD statistic r^2^ > 0.2, resulting in the identification of a total of 16 QTLs (**Table 2**). Notably, significant differences were observed in the Vc content of materials with different alleles at the peak positions of the four related loci (*qVc.A01-1*, *qVc.C01-2*, *qVc.C01-3*, and *qVc.C01-4*). For *qVc.A01-1*, the analysis revealed that the two variant bases, G and A, associated with the peak SNP seq-new-rs32414 were present at frequencies of 92% and 8%, respectively. The average Vc content for the A allele (102.0 mg/100g) was significantly higher than that for the G allele (96.5 mg/100g), with P = 2.0E−4 (**Figure 3A**). For the locus *qVc.C01-2*, the peak SNP seq-new-rs30616 indicated that the two variant bases A and T represented 77% and 23%, respectively. The mean Vc content of the T allele (98.4 mg/100g) was significantly higher than that of the A allele (96.4 mg/100g), with P = 0.021 (**Figure 3B**). For *qVc.C01-3*, the peak SNP Bn-scaff_16809_1-p69403 had two variant bases, A and G, comprising 74% and 26% respectively. The average Vc content of the A allele (97.8 mg/100g) was significantly higher compared to that of the G allele (95.6 mg/100g), with a P-value of 0.027 (**Figure 3C**). For *qVc.C01-4*, the peak seq-new-rs38261 had variant bases G and A, contributing 81% and 19%, respectively. The average Vc content for the G allele (98.6 mg/100g) was significantly higher than that for the A allele (95.8 mg/100g), with a P-value of 0.019 (**Figure 3D**). Thus, the SNP loci affecting Vc content in rapeseed may serve as potential markers for breeding, pending further validation.

**Table 2:** The details of 16 association loci for Vc content under different environmental conditions.

QTLs	Physical interval (bp)	Peak SNP	Chr	Position	Environment	-log_10_(*p*)	r2 (%)
*qVc.A01-1*	10,924,975- 11,981,733	seq-new-rs25149	A01	11,424,975	YL2018, WC2019, WC2020, BLUP	4.60-5.61	6.88-8.09
*qVc.A01-2*	19,965,174- 20,965,174	Bn-A01-p24697185	A01	20,465,174	YL2019	4.68	6.74
*qVc.A02*	2,072,188- 3,072,188	seq-new-rs38874	A02	2,572,188	YL2020	4.81	7.11
*qVc.A03*	15,243,323- 16,243,323	seq-new-rs25559	A03	15,743,323	WC2020	4.40	6.34
*qVc.A06*	21,756,831- 23,018,960	Bn-A06-p23542209	A06	22,518,960	YL2020	4.97-5.19	6.46-7.24
*qVc.A10*	10,640,905- 11,640,905	Bn-A10-p9740870	A10	11,140,905	YL2020	4.46	6.53
*qVc.C01-1*	7,710,474- 8,710,474	seq-new-rs46108	C01	8,210,474	YL2020, BLUP	4.41-5.25	6.72-7.58
*qVc.C01-2*	11,219,801- 12,859,994	seq-new-rs30616	C01	11,858,918	YL2018, YL2020, BLUP	4.43-6.08	6.34-9.34
*qVc.C01-3*	18,064,125- 20,955,252	Bn-scaff_16809_1-p69403	C01	18,564,125	YL2018, YL2019, BLUP	4.37-5.98	6.58-9.71
*qVc.C01-4*	24,407,771- 25,407,771	seq-new-rs38261	C01	24,907,771	YL2018, YL2019, WC2019, YL2020, WC2020, BLUP	4.44-9.26	6.50-12.78
*qVc.C01-5*	31,668,263- 32,668,263	seq-new-rs35180	C01	32,168,263	WC2019, BLUP	4.35-5.01	6.18-7.26
*qVc.C01-6*	37,878,354- 38,878,462	seq-new-rs38269	C01	38,378,354	JZ2018	4.44-4.53	8.07-8.18
*qVc.C02*	17,238,926- 19,094,998	Bn-scaff_16369_1-p182886	C02	18,593,794	YL2019	4.62-5.53	5.82-7.11
*qVc.C03*	21,787,311- 23,751,769	Bn-scaff_17298_1-p1136461	C03	23,251,769	BLUP	4.34-4.52	6.19-6.41
*qVc.C05*	14,141,465- 15,141,465	seq-new-rs40830	C05	14,641,465	YL2018, YL2019, WC2019, YL2020, WC2020, BLUP	4.36-9.17	6.49-12.76
*qVc.C06*	21,940,239- 22,476,039	Bn-scaff_15818_1-p2532249	C06	21,940,239	YL2020	4.60-4.94	5.76-6.12

### Functional annotation and enrichment analysis of SNP-associated genes

**Figure 4:**
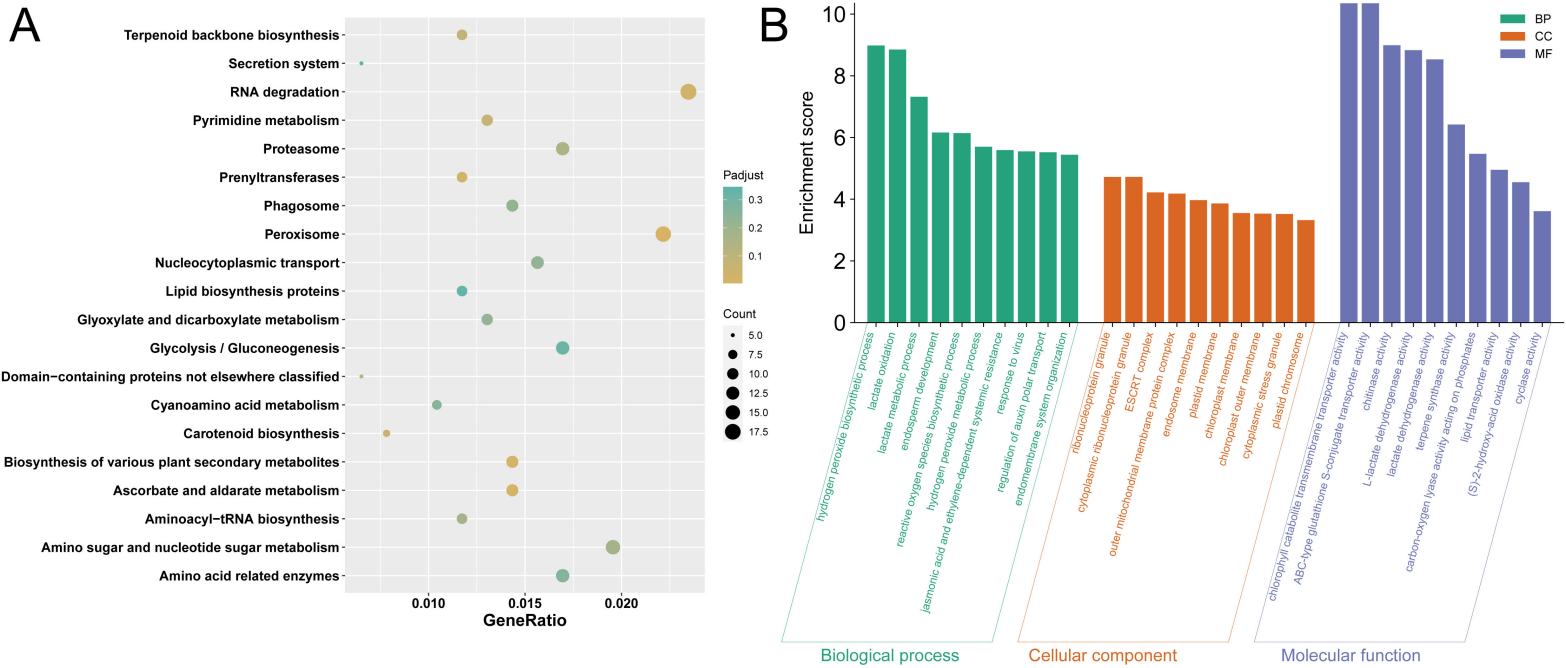
KEGG and GO cluster pathway analysis of candidate genes. **(A)** Dot plot of the KEGG pathway enrichment. The horizontal axis represents the enrichment rate of the input genes in the pathway, while the vertical axis represents the pathway name. The color scale indicates different thresholds of the p value, and the size of the dot indicates the number of genes corresponding to each term. **(B)** GO function analysis histogram. Biological Process (BP) is marked by dark cyan, Cellular Component (CC) is marked by sienna, and Molecular Function (MF) is marked by steel blue.

Additionally, concerning the primary SNPs associated with these loci, we identified a total of 2365 annotated genes within a 500 kb range (both upstream and downstream) (**Supplementary Table S4**). To further elucidate the functional implications of these annotated genes, we conducted KEGG and GO enrichment analyses using the BnTIR website (https://yanglab.hzau.edu.cn/), respectively. The results indicated significant enrichment of the annotated genes in various KEGG pathways, such as peroxisome metabolism, ascorbate and aldarate pathways, biosynthetic processes of different plant secondary metabolites, prenyltransferase functions, RNA degradation pathways, carotenoid biosynthesis, pyrimidine metabolism, and terpenoid backbone biosynthesis (**Figure 4A**). For the GO enrichment terms, the top ten from each category were organized according to “-log_10_ (*p*)” values, listed from smallest to largest, and illustrated in a histogram (**Figure 4B**). In the biological process category, the majority of annotated genes were primarily linked to the hydrogen peroxide biosynthetic process, lactate oxidation, lactate metabolic process, endosperm development, reactive oxygen species biosynthetic process, and hydrogen peroxide metabolic process. Within the cellular component category, most annotated genes were associated with ribonucleoprotein granules, cytoplasmic ribonucleoprotein granules, ESCRT complexes, outer mitochondrial membrane protein complexes, endosome membranes, plastid membranes, and chloroplast membranes. As for the molecular function category, these annotated genes were predominantly associated with chlorophyll catabolite transmembrane transporter activity, ABC-type glutathione S-conjugate transporter activity, chitinase activity, and L-lactate dehydrogenase activity.

### Prediction of Vc candidate genes in rapeseed seedlings

**Figure 5:**
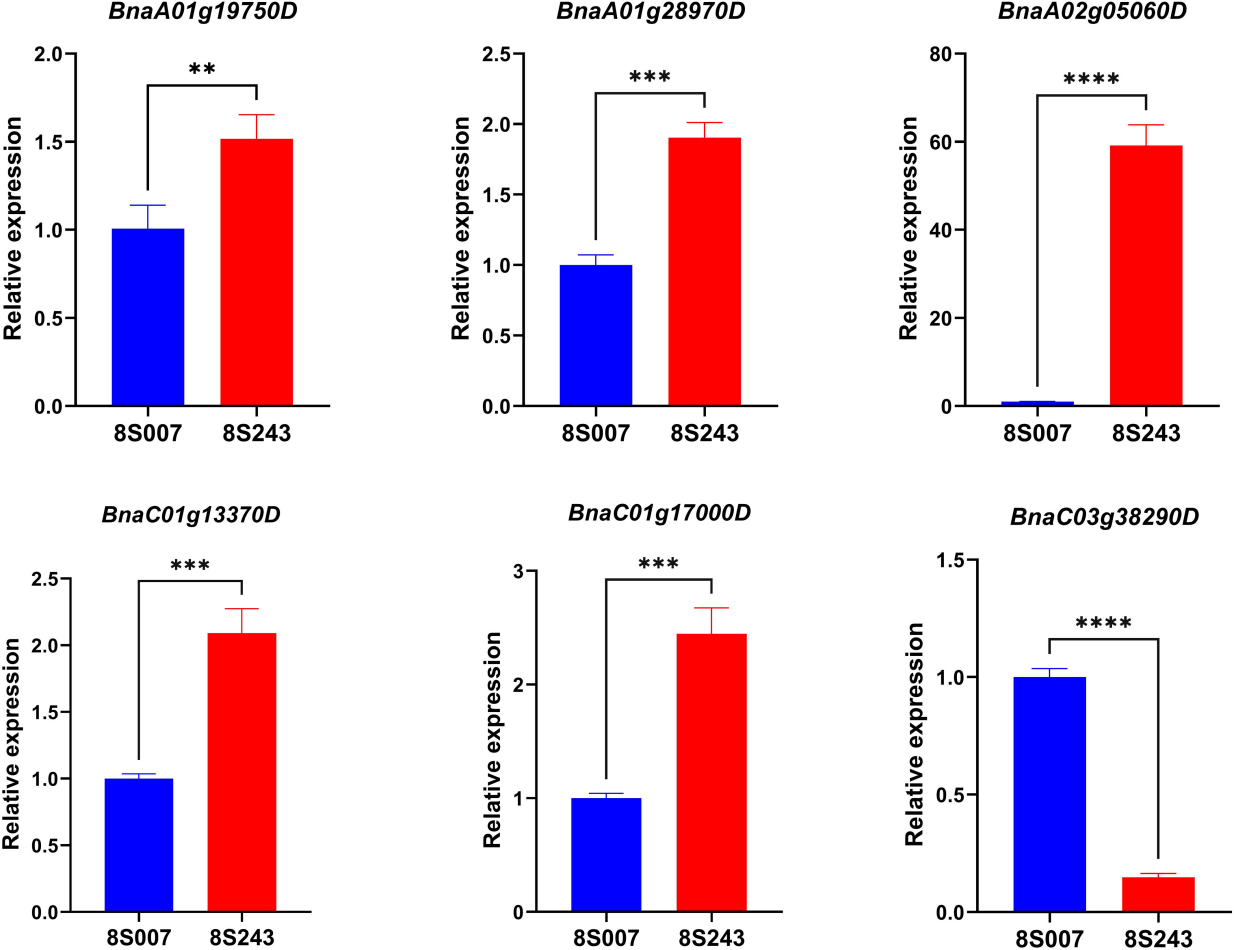
The six candidate genes expression level rapeseed leaves of two accessions (8S007 and 8S243) of based on qRT-PCR. **, ***, and **** indicate statistically significant differences, with a P-value threshold of less than 0.05, 0.001, and 0.001 respectively, as assessed through Student’s t-test.

Within the 16 QTLs associated with Vc content in rapeseed seedlings, we successfully identified 16 candidate genes based on the functional annotations of homologous genes in *Arabidopsis* (**Supplementary Table S5**). We assessed the expression levels of these 16 candidate genes in the leaves of two accessions that exhibited significant variations in Vc content using qRT-PCR analysis. The qRT-PCR analysis revealed distinct expression patterns among the candidate genes. Specifically, six genes exhibited significant variation in expression levels between the high-Vc accession (8S243) and low-Vc accessions (8S007), as illustrated in **Figure 5**. Notably, the expression levels of *BnaA01g19750D*, *BnaA01g28970D*, *BnaA02g05060D*, *BnaC01g13370D*, and *BnaC01g17000D* were significantly higher in the high-Vc accession (8S243) compared to the low-Vc accession (8S007). Conversely, *BnaC03g38290D* showed significantly higher expression levels in the low-Vc accession (8S007) than in the high-Vc accession (8S243). Within the QTL *qVc.A01-1*, *BnaA01g19750D* (a homolog of *At3g50820*) was selected as the key candidate gene, which is essential for photosynthesis, particularly in the function and stability of photosystem II (Popelkova and Yocum, 2011; Kakiuchi et al., 2012). Previous research has shown that there is a direct connection between Vc biosynthesis and photosynthesis (Zha et al., 2019; Mellidou et al., 2021). Additionally, *BnaA01g28970D* (a homolog of *At3g15210*), located in *qVc.A01-2*, is annotated in the *Arabidopsis* as ethylene-responsive element binding factor 4 (ERF4). For example, the *Arabidopsis* gene *AtERF98* promotes the transcriptional activation of Vc synthesis to enhance salt tolerance (Zhang et al., 2012). In the QTL *qVc.A02*, the candidate gene *BnaA02g05060D* was identified as a homolog of *At5g20720* (CHAPERONIN 20, CPN20). Research has shown that the CPN20 is crucial for the development and function of chloroplasts in *Arabidopsis* by interacting with FSD1 and regulating its activity, as well as affecting FeSOD activity (Kuo et al., 2012). Another significant candidate gene, *BnaC01g13370D*, identified as a homolog of *At4g21960*, is located within the *qVc.C01–1* locus. Peroxidase plays a vital role in the oxidative stress response and metabolic pathways in plants by eliminating excess reactive oxygen species (ROS) (Lazzarotto et al., 2015; Choudhury et al., 2016). In the QTLs of *qVc.C01-2*, the candidate gene *BnaC01g17000D* was identified as a homolog of *At4g25080*. The enzyme encoded by the *CHLM* (*At4g25080*) gene is a rate-limiting factor in the chlorophyll biosynthesis pathway (Wang et al., 2017). Within the QTL *qVc.C03*, the candidate gene *BnaC03g38290D* was identified as a homolog of *At3g14110* (*FLU*). The *FLU* gene is crucial for regulating the synthesis of chlorophyll, photosynthesis, and response to light changes in plants (Hou et al., 2018). These findings suggest that the identified genes with differential expression may play crucial roles in regulating Vc accumulation in rapeseed seedlings. Further functional validation of these candidate genes through genetic and biochemical approaches will be essential to elucidate their specific contributions to Vc biosynthesis and accumulation pathways.

### Selection of superior germplasm resources

**Figure 6:**
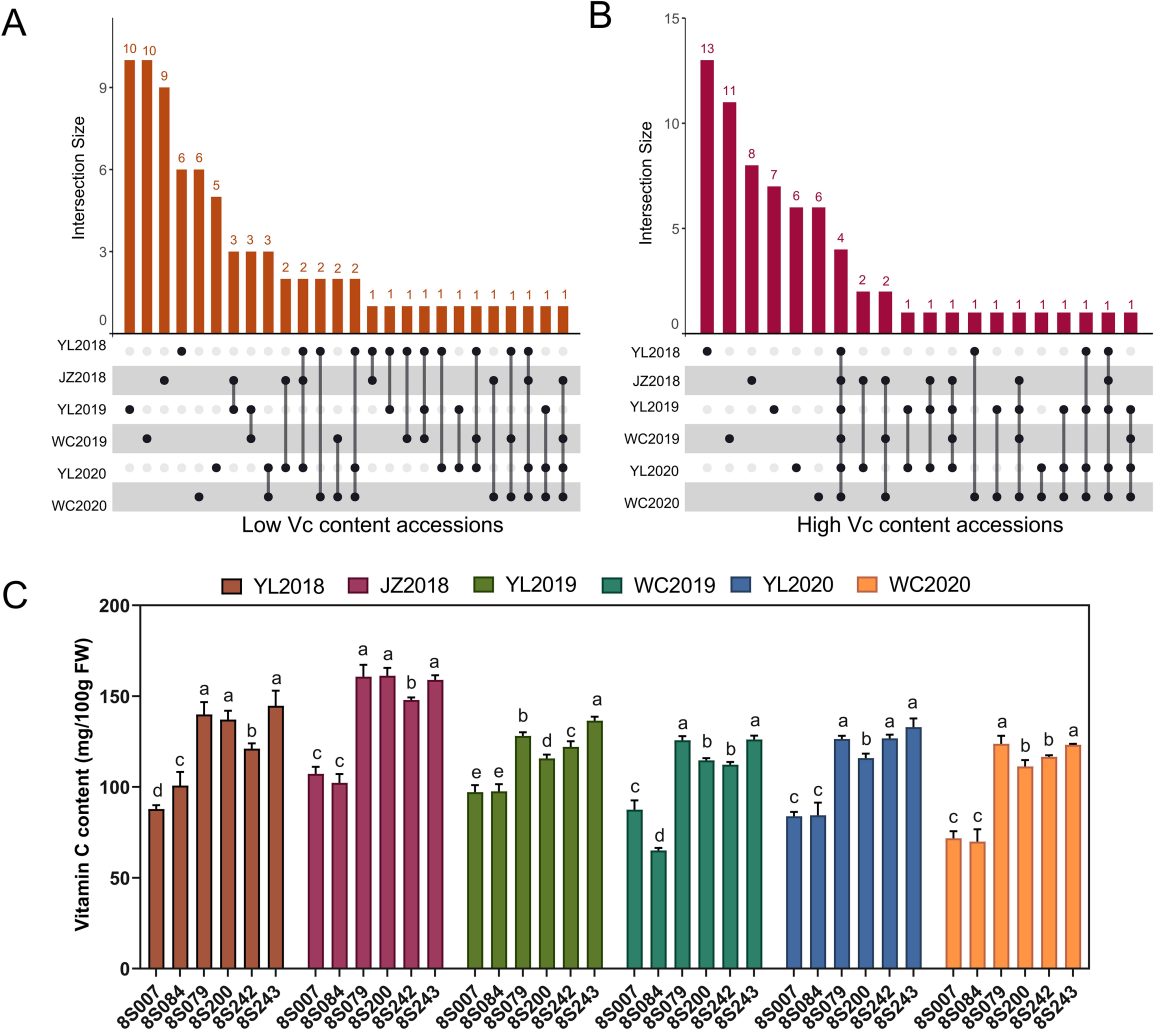
Statistical analysis of high and low Vc germplasms for six environments. **(A)** UpSet diagram showing low Vc content accessions for six environments. **(B)** UpSet diagram showing high Vc content accessions for six environments. **(C)** The statistical analysis of Vc content in two low-Vc and four high-Vc accessions for six environments. In figures, the different letters above the bars denote significance groupings (*P* < 0.05) as determined by ANOVA.

The statistical analysis conducted led to the identification of the top 20 accessions based on their Vc content, specifically highlighting both the highest and lowest values observed across various environmental conditions (**Supplementary Table S6**). Among the top 20 accessions with low Vc content, the average Vc content across the six environments ranges from 69.75 to 105.27 mg/100g. Similarly, the average Vc content for among the top 20 accessions with high Vc content ranges from 101.03 to 141.11 mg/100g across the six environments. Intersection analysis was used to analyze extreme Vc germplasms screened from the six environments. Notably, among the top 20 low Vc accessions across different environments, two accessions were consistently identified in four environments (**Figure 6A**). Among them, 8S007 exhibited low Vc content levels in YL2018 (87.84 mg/100g), JZ2018 (107.23 mg/100g), YL2020 (83.90 mg/100g), and WC2020 (71.71 mg/100g) (**Figure 6C**). Similarly, 8S084 demonstrated low Vc content in JZ2018 (102.23 mg/100g), WC2019 (65.01 mg/100g), YL2020 (84.39 mg/100g), and WC2020 (69.89 mg/100g). Additionally, among the top 20 high Vc accessions across different environments, four accessions were consistently identified in all six environments (**Figure 6B**). These accessions, namely 8S079, 8S200, 8S242, and 8S243, were recognized as exceptional accessions that could stably exhibit high Vc content across six environments. Notably, in the 2018JZ environment, these four accessions exhibited the highest Vc content, with values of 160.72 mg/100g, 161.25 mg/100g, 147.84 mg/100g, and 158.91 mg/100g, respectively (**Figure 6D**). In summary, these findings highlight not only the genetic diversity in Vc content among accessions but also the potential of these select accessions as valuable germplasm resources.

## Discussion

### Genetic mapping reveals the complex genetic basis of vitamin C content in rapeseed

Rapeseed seedlings, recognized as a novel and nutritious fresh vegetable, hold significant potential for future applications in the processing and utilization of multifunctional vegetables. Our previous studies have indicated that the levels of Vc in rapeseed seedlings and flower stalks exceed those found in other typical vegetables. However, the genetic factors influencing Vc content in rapeseed remain inadequately explored, leading to a limited availability of superior germplasm resources. To investigate the genetic basis of Vc content in rapeseed seedlings, this study conducted a GWAS and candidate gene analysis, establishing a foundation for subsequent research on Vc content in rapeseed. Studies have shown that Vc content is affected by various genes, characterizing it as a quantitative trait with a complex genetic system. The phenotypic variation in Vc content among 327 rapeseed seedlings accessions across six different environments demonstrates the complexity of this quantitative trait (**Figure 1**). The normal distribution of Vc content in rapeseed seedlings suggests that it is polygenic, controlled by multiple genes, and therefore suitable for GWAS analysis. The genetic basis of Vc has been identified in various plants, including *Arabidopsis* (Zhang et al., 2009), tomato (Chen et al., 2020), kiwifruit (Chen et al., 2021), apple (Davey et al., 2006), strawberry (Zorrilla-Fontanesi et al., 2011) and others. In our study, we used the MLM model to conduct GWAS on the association population and identified 31 significantly associated SNPs distributed across various chromosomes (**Figure 2**). These SNPs accounted for a notable proportion of the phenotypic variation (5.68% to 12.78%), indicating their potential role in the regulation of Vc content (**Supplementary Table S2**). To our knowledge, the present research represents the inaugural study on QTL mapping concerning Vc content in rapeseed seedlings. By integrating notable SNP loci, we identified a total of 16 QTLs. In addition, the allele-specific effects on Vc content, as demonstrated by the mean Vc content differences associated with specific alleles only at *qVc.A01-1*, *qVc.C01-2*, *qVc.C01-3*, and *qVc.C01-4*, provide important insights into the genetic framework of Vc content in rapeseed seedlings (**Figure 3**). Several potential reasons may account for this phenomenon. On the one hand, Vc content is a quantitative trait regulated by multiple genes, and no significant QTLs related to Vc have been previously identified. On the other hand, the process of vitamin C biosynthesis is complex and influenced by various factors including light conditions, temperature variations, and the presence of plant hormones (Sami et al., 2024). The Vc-related QTLs and SNPs identified in this study were not compared with results from other research due to a lack of related studies. Therefore, to validate the findings of the current research, it is essential to conduct multi-batch experiments in greenhouses in the future to mitigate the impact of environmental factors.

### Candidate gene mining and exploration of potential regulatory mechanisms

While previous research has identified potential genes associated with Vc levels in various crops, there has been limited discovery of genes that control Vc content specifically in rapeseed seedlings. In this study, we identified 16 candidate genes within 16 QTLs associated with Vc content through screening (**Supplementary Table S5**). These genes, which have homologs in *Arabidopsis* with known functions in photosynthesis and Vc biosynthesis, are strong candidates for further functional verification. Additionally, Vc levels are influenced by both internal triggers and external conditions, with light being a key factor (Paciolla et al., 2019). The expression levels of six candidate genes in the leaves of two accessions (8S007 and 8S243) were significantly confirmed by qRT-PCR analysis (**Figure 5**). Among them, the annotation of four genes and their corresponding studies in species such as *Arabidopsis thaliana* have indicated connections to biological processes such as photosynthesis. Therefore, we speculate that light and related biological processes play significant roles in the biosynthesis of rapeseed. Previous research has indicated that the synthesis of Vc is dependent on light, with levels increasing upon exposure to light, decreasing in darkness, and rising further under high-light conditions (Mastropasqua et al., 2012). The key candidate gene was selected *BnaA01g19750D*, a homolog of *At3g50820* (*PSBO2*). This gene is essential for photosynthesis, particularly in the function and stability of photosystem II (Popelkova and Yocum, 2011; Kakiuchi et al., 2012). Under high light conditions, the function of *PSBO2* is particularly important, the leaf weight of the *psbo2* mutant was notably diminished, indicating that *PSBO2* is vital for maintaining photosynthetic efficiency (Duchoslav and Fischer, 2015). Another candidate gene, *BnaA02g05060D*, is a homolog of *At5g20720* (*CPN20*). This gene is almost not expressed in the 8S007 accession, while its expression level in the 8S243 accession is approximately 80 times higher than that of the low Vc accession. Studies have demonstrated that *CPN20* interacts with the molecular chaperone *CPN60* to facilitate protein folding, a function that is critical for the proper folding and functionality of proteins within the chloroplast, as most proteins in the chloroplast are directly or indirectly involved in photosynthesis (Wang et al., 2021). The candidate gene *BnaC01g17000D* has been identified as a homolog of *At4g25080*. Previous studies have demonstrated that *CHLM* plays a crucial role in the biosynthesis of chlorophyll, the development of chloroplasts, and the signaling between plastids and the nucleus. Additionally, The function of the *CHLM* gene also involves interactions with other genes related to chlorophyll synthesis and photosynthesis (Huang et al., 2020).

We have identified four differentially expressed candidate genes in high- and low-Vc materials, all of which are involved in photosynthesis. The carbohydrates produced through photosynthesis are ultimately stored in the form of sugars, which significantly impact various stages of the plant life cycle and can be converted into fructose and glucose in storage tissues (Dou et al., 2022). We speculate that these candidate genes may influence the synthesis of the final photosynthetic products, which are sugars. Glucose serves as indeed the primary carbon source and starting point for Vc biosynthesis via the L-galactose pathway, which is the dominant route in plants, algae, and many protists (Feng et al., 2021). Glucose provides the entire carbon skeleton that is progressively modified through enzymatic reactions to ultimately form the L-galactose structure needed for Vc. Our previous studies, based on transcriptome analysis, metabolite profiling, and precursor substance treatments, have demonstrated that the L-galactose pathway is the primary route for Vc biosynthesis in rapeseed (Wang et al., 2025). However, the molecular pathway involved in the synthesis of Vc in rapeseed remains ambiguous, necessitating validation of the roles of candidate genes in future research. Although no homologous genes related to Vc biosynthesis have been reported among the identified candidate genes, they may indirectly regulate known genes in the Vc synthesis pathway. The synthesis of Vc in rapeseed likely involves a collaborative process engaging several genes and cannot be solely attributed to a single gene. The candidate genes and loci detected in this study provide valuable insights for the future understanding of the genetic mechanisms underlying Vc content in rapeseed. Through the cloning of key genes associated with Vc content in rapeseed, the development of molecular markers, and the analysis of the underlying molecular mechanisms of anabolism, we aim to provide theoretical, material, and technical support for the molecular breeding of rapeseed with high Vc content.

### Identification of elite germplasm resources and breeding application value

In the context of enhancing the nutritional quality of rapeseed, the strategic utilization of elite germplasm resources emerges as a pivotal approach. The scarcity of germplasm with high Vc content in rapeseed underscores the significance of the findings presented in this study. By selecting the top 20 accessions with extreme Vc content from diverse environments, this selection not only identifies accessions with stable high or low Vc content across multiple environments but also enriches the genetic pool available for breeding programs. The recurrent identification of certain accessions among the top performers, including the four high Vc germplasms (8S079, 8S200, 8S242, and 8S243) recognized across six environments (**Figure 6**), suggests their potential as parental accessions for developing high Vc rapeseed varieties. The average Vc content of these four excellent germplasm resources grown in different environments was 134.08, 125.98, 124.43, and 137.06 mg/100g, respectively. Among them, the Vc content of 8S079 and 8S243 demonstrates higher Vc levels than the vast majority of *Brassica* vegetables currently on the market (Domínguez-Perles et al., 2014). Notably, the recommended dietary intakes (RDAs) of Vc vary across different regions of the world. Among these, the European Food Safety Authority (EFSA) has increased the Vc RDAs to 110 mg and 95 mg every day for men and women, respectively (Carr and Lykkesfeldt, 2020). Individuals can easily satisfy their daily Vc needs by consuming less than 100 grams of fresh rapeseed seedlings, such as the 8S079 and 8S243 germplasms. The consistently high Vc levels maintained by these accessions underscore their value in breeding programs aimed at enhancing the nutritional value of vegetable-rapeseed. Their stable performance indicates a reliable genetic basis for Vc content, which is essential for creating new varieties. Incorporating these accessions as parental lines in breeding crosses could facilitate the development of new rapeseed varieties with improved Vc content. This enhancement not only addresses the current limitations in high Vc germplasm resources but also contributes to the broader goal of enriching the nutritional profile of vegetable-rapeseed. Furthermore, we have identified two low-Vc rapeseed germplasms (8S007 and 8S084) that demonstrate relative stability across different environments. Subsequently, we can hybridize these high and low extreme germplasms and materials to construct an F_2_ population, screen for extreme materials, and utilize Bulk Segregant Analysis (BSA) to identify and regulate the primary QTL site associated with Vc synthesis. Additionally, transcriptomic and metabolomic analyses can be conducted on the extreme materials, facilitating a multi-omics approach to investigate the genetic mechanisms underlying the biosynthesis of Vc in rapeseed.

## Conclusions

In conclusion, this study enhances our understanding of the genetic regulation of Vc content in rapeseed. Through GWAS and candidate gene analysis, we identified 31 SNPs and 16 QTLs associated with Vc accumulation, highlighting the polygenic basis of this trait. The identified candidate genes, which have homologs in *Arabidopsis* involved in photosynthesis and Vc biosynthesis, present promising targets for further functional verification. These findings establish a foundation for understanding the genetic mechanisms underlying Vc synthesis in rapeseed and pave the way for molecular breeding efforts aimed at enhancing its nutritional value. By selecting top-performing accessions with consistently high Vc content, we have enriched the genetic pool for breeding programs. Accessions such as 8S079 and 8S243 not only exhibit superior Vc levels but also meet the recommended dietary intakes, making them valuable for the development of high-Vc rapeseed varieties. The integration of these genetic resources, along with ongoing research into candidate gene functions, will facilitate the creation of rapeseed with improved nutritional profiles, contributing to healthier and more sustainable vegetable-rapeseed production.

## Data Availability

The raw data supporting the conclusions of this article will be made available by the authors, without undue reservation.
